# Gut bacteriome alterations during high altitude exposure: a comprehensive analysis of different species

**DOI:** 10.3389/fmicb.2026.1762563

**Published:** 2026-02-18

**Authors:** Xinxin Yin, Jin Zeng, Zhiqi Li, Siai Chen, Changpeng Xie, Xuejing Li, Liping Zhang, Yuanming Pan, Juan An

**Affiliations:** 1 Department of Basic Medical Sciences, Qinghai University Medical College, Xining, Qinghai, China; 2 Cancer Research Center, Beijing Chest Hospital, Capital Medical University/Beijing Tuberculosis and Thoracic Tumor Research Institute, Beijing, China

**Keywords:** altitude hypoxia, environmental adaption, gut bacteriome, microbial diversity, microbiome

## Abstract

With the increase of high-altitude sojourn population, more and more studies on hypoxia have been conducted, but the associated changes in gut bacteriome in different hypoxic environments need to be further investigated. Gut bacteriome plays an important role in host adaptation to high-altitude hypoxia, but the contribution of gut bacteriome in high-altitude hypoxia adaptation is still controversial and may be influenced by multiple factors. In this study, we reviewed the changes in diversity and composition of the gut bacteriome of different populations of animals in different highland hypoxic environments, clarified the dynamics of the gut bacteriome during exposure to high altitude, identified the core bacteriome that may contribute to host adaptation to hypoxic environments, and comprehensively considered the effects of multiple factors on the gut bacteriome.

## 1 Introduction

Oxygen is necessary for the maintenance of human metabolism and physiological functions. The cold hypoxia, high wind speeds and strong ultraviolet rays that characterize the plateau region can cause an adverse multi-system stress response in the body. Plateau reaction, also known as acute mountain sickness (AMS), is a series of plateau-associated illnesses caused by the body’s difficulty in adapting to the hypoxic environment due to the decrease in oxygen concentration in the atmospheric pressure and the decrease in the partial pressure of oxygen after the body enters a certain altitude (Luks et al., 2017; Garrido et al., 2021; Gupta et al., 2024).

**FIGURE 1:**
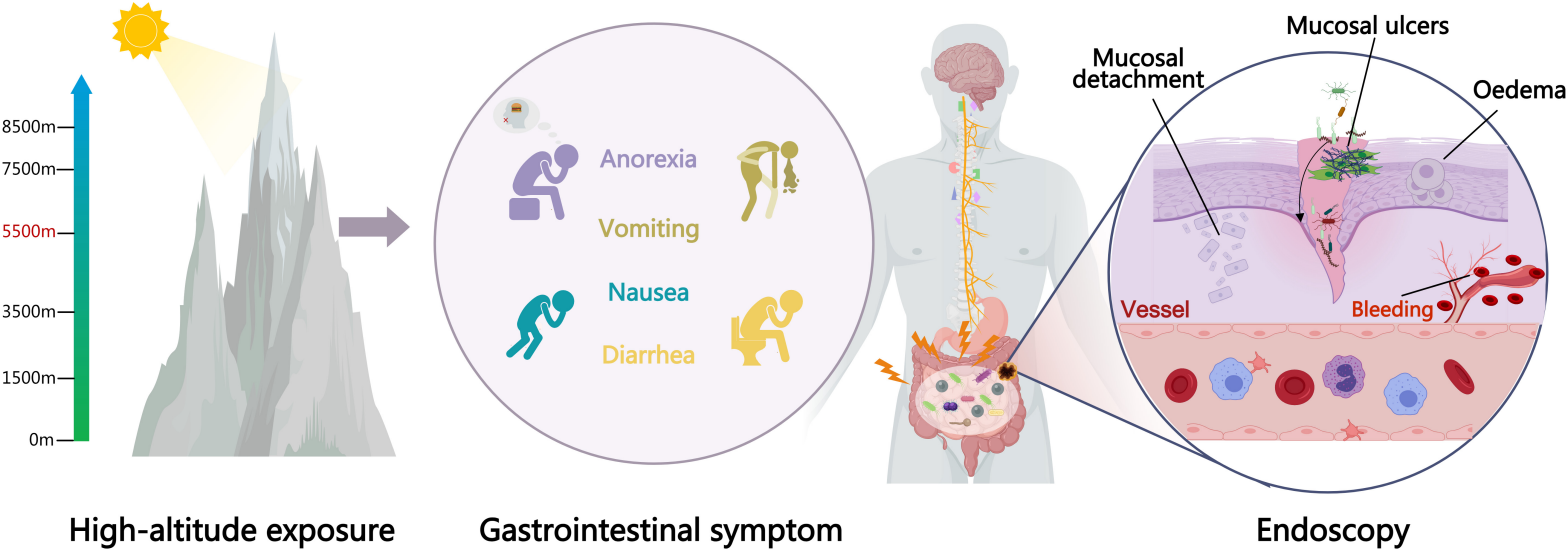
Gastrointestinal stress caused by high altitude exposure affects the intestinal bacteriome, and disruption of the intestinal bacteriome accelerates the body’s damage.

The gastrointestinal tract is also a key organ in the development of AMS, and the body may experience a variety of gastrointestinal stress symptoms such as loss of appetite or nausea, vomiting and incapacitation when traveling at high altitude. Approximately 80 per cent of people with acute mountain sickness (AMS) report at least one gastrointestinal symptom (e.g., anorexia, nausea, diarrhea, vomiting, etc.) (Luks et al., 2017; McKenna et al., 2022a,^2023^; Chen et al., 2024; Figure 1). One of the main functions of the intestine is to regulate the transport of water, electrolytes and nutrients. To maintain these functions, the epithelium of the intestine is in close contact with the gastrointestinal lumen. As the lumen is connected to the external environment, which may have a high bacterial and antigenic load, the epithelium must also prevent pathogens in the gastrointestinal lumen from entering the internal tissues (Shen, 2009; Di Vincenzo et al., 2024; Macura et al., 2024; Nwako et al., 2025; Figure 1). Hypoxia induces severe primary intestinal barrier dysfunction, promotes bacterial and endotoxin translocation, and causes systemic inflammatory responses; it is a major factor in plateau multiorgan dysfunction syndrome (Manresa and Taylor, 2017; Kim et al., 2020; McKenna et al., 2022b). In the study of the complex physiological functions of the intestinal tract, we note that the intestinal tract is not only an important organ for digestion and absorption of nutrients, but also has the functions of immunoregulation, endocrine and mucosal barrier. The intestinal mucosal barrier function is an important part of the body barrier system, which has been valued by many researchers. The intestinal mucosal barrier consists of mechanical barrier, immune barrier, chemical barrier and biological barrier (An et al., 2022). Gut bacteriome plays an important role in maintaining the microecological balance of the gastrointestinal tract in various animals (Halverson and Alagiakrishnan, 2025). In addition to this, disturbances in the intestinal bacteriome may lead to the proliferation of potentially pathogenic bacteria, allowing pathogenic bacteria to become dominant, leading to intestinal immune dysfunction, intestinal inflammation and other intestinal diseases (Paul et al., 2025). Numerous studies have demonstrated that high altitude exposure affects the normal function of the gut and the composition of the gut bacteriome (Su et al., 2024; Chen Q. et al., 2025; Qi et al., 2025; Figure 1). Therefore, the hypoxic environment at high altitudes not only induces widespread physiological changes and triggers a series of altitude sickness symptoms, but may also lead to severe diarrhea and intestinal barrier damage. This can subsequently cause bacterial translocation and systemic or localized inflammatory responses. This reaction develops rapidly, has a short duration, and can be life-threatening in severe cases (Zhang et al., 2022).

The gut harbors a rich and diverse array of bacteria. The intestine is mainly colonized by four bacterial phyla: *Firmicutes*, *Bacteroidetes*, *Proteobacteria*, and *Actinobacteria* (Bibbò et al., 2017). The use of antibiotics, diet and pH value can interfere with the gut bacteriome, and the alteration of the gut bacteriome (dysbiosis) can induce abnormal immune responses in the gut-associated lymphoid tissue (GALT), and these changes can also impair the systemic immune response (Pabst and Mowat, 2012; Gomes et al., 2018; Botía-Sánchez et al., 2021). The gut bacteriome regulates the host immune response through two main mechanisms: activation of the innate immune response through Toll-like receptors (TLR) (Xie et al., 2018) and/or activation of Free Fatty Acid Receptors (FFAR) via microbial metabolites such as Short Chain Fatty Acids (SCFAs), including acetate, propionate and butyrate (Huang et al., 2021). In addition, these metabolites can induce initial T cells to differentiate into regulatory T cells (Treg) or migrate into the gut (Scott et al., 2018). Dysbiosis of the gut bacteriome can lead to over-activation of TLR and low SCFAs production, leading to the development of many gastrointestinal disorders, obesity and diabetes (Fava and Danese, 2011). Additionally, the gut bacteriome and its metabolites exert certain effects on the differentiation and function of adaptive immune cells. They promote the differentiation of naive T cells into regulatory T cells (Tregs) with immunosuppressive functions, thereby maintaining intestinal immune tolerance (Zheng et al., 2020; Wang et al., 2023b; Shi et al., 2025; Brown et al., 2026). Simultaneously, under specific conditions, they can induce the differentiation of pro-inflammatory Th17 cells and other subsets, thus balancing defense and tolerance. By stimulating gut-associated lymphoid tissue (GALT), the bacteriome promotes the production of secretory immunoglobulin A (SIgA), thereby preventing pathogen adhesion and translocation through the epithelial layer (Zheng et al., 2020; Ullah et al., 2024; Gu et al., 2025). This process plays a central role in maintaining microbial homeostasis and defending against infection.

Exposure to low-pressure hypoxic environments alters gut bacteriome. Across different species, high-altitude hypoxia consistently disrupts gut communities, yet the specific manifestations of these changes vary significantly—not only between species but also within the same species. The duration of exposure further modulates the nature of these bacteriome alterations. Consequently, While we understand that high altitude affects the gut bacteriome, these effects are not universal and vary across species, populations, or ecological systems. A key unresolved question remains: Are these bacteriome community alterations merely secondary reflections of host physiological states, or do they actively participate in the pathogenesis of altitude-related diseases? However, a large number of studies suggest that the gut bacteriome may be a causative factor in high altitude-related pathogenesis (Wang F. et al., 2022; Sun et al., 2025) and a target for therapeutic intervention (Chen Q. et al., 2025). Therefore, a more detailed elucidation of the alterations in the gut bacteriome after low-pressure hypoxia exposure at plateau is particularly important for the subsequent pathogenesis and therapeutic intervention of high altitude-related diseases.

## 2 Effect of low oxygen environment in highland on the diversity of gut bacteriome of different organisms

### 2.1 Effects of acute plateau hypoxia on the diversity of animal gut bacteriome

Rapid ascent to high altitudes can cause low-pressure hypoxia, triggering a series of altitude sickness symptoms. Research on acute high-altitude hypoxia has primarily focused on laboratory animals, typically within a 1-month timeframe. Studies indicate that following 24 h of simulated hypoxic exposure at an altitude of 5,500 m, C57BL/6J mice exhibit a significant increase in α diversity (Wang F. et al., 2022). In our study of BALB/c mice, exposure to 6,000 m altitude for 7 days resulted in a significant increase in gut bacteriome α diversity compared to low-altitude conditions (Zeng et al., 2025). In another study involving BALB/c mice exposed to 5,000 m altitude for 30 days, no significant difference in α diversity was observed (Zhang et al., 2018a). In contrast, in Wang Y. et al.’s (2022) studies indicate that following 4 weeks of simulated high-altitude hypoxia exposure at 4,000 m, C57BL/6J mice exhibit significantly increased α diversity, which is in alignment with Zhao et al. (2022), Zhang et al. (2023a), and Li et al. (2025). Meanwhile, in a study examining fecal dynamics in rats subjected to simulated high-altitude conditions at 5,000 m during 28 days of low-pressure hypoxia, Pan et al. (2022) found no significant changes in α diversity during the initial 28 days of low-pressure hypoxia, consistent with the findings of Zhang et al. (2018a), Wang et al. (2023a), but α-diversity was significantly increased on day 28. In addition, in two studies on the dynamic bacteriome of plateau hypoxic SD rats (Sprague Dawley Rat), it was found that the α-diversity of their gut bacteriome was affected differently with time, and Han et al. (2022) showed that the α-diversity fluctuated significantly in the first 5 days of the hypoxic treatment, and then the α-diversity showed an increasing trend, and then gradually decreased after 4 weeks, and also showed a significant fluctuation in the first 5 days of the resumption of normoxic treatment, and then gradually increased, and then gradually increased after 4 weeks, and also fluctuated during the first 5 days after the return to normoxia. Furthermore, based on PCoA analysis, microbial structures showed distinct separation with variations in oxygen concentration and atmospheric pressure (Han et al., 2022). Meanwhile, the β-diversity of gut microorganisms showed significant differences in a large number of studies.

It is evident that the impact of acute high-altitude hypoxia exposure on the gut bacteriome involves a dynamic and complex process (Pan et al., 2022), with its specific manifestations potentially influenced by multiple interacting factors. First, significant variations in hypoxia exposure duration and altitude across different studies directly affect the severity and pattern of hypoxia experienced by the organism, potentially leading to distinct stress and adaptive responses within the bacteriome. Second, during the initial acclimatization phase, animals commonly exhibit reduced locomotor activity (Alvarez-Araos et al., 2024) and decreased appetite (Barclay et al., 2023). These changes indirectly alter nutrient intake, gastrointestinal motility, and metabolic states, further disrupting the composition and function of the gut bacteriome (McKenna et al., 2022b). Additionally, variations in species and individual genetic backgrounds determine inconsistent adaptability to high-altitude environments. This biological diversity likely drives divergent microbial responses. Therefore, we hypothesize that the conflicting results observed in existing studies are closely linked to factors such as exposure conditions, behavioral changes, and host genetic heterogeneity.

### 2.2 Effects of long-term highland hypoxic environment on the diversity of animal gut bacteriome

Research on wild animals chronically exposed (>1 month) to high-altitude environments reveals significant shifts in gut bacteriome diversity compared to lowland counterparts, though findings on α-diversity are not uniform. Classified as follows:

Increased gut bacteriome diversity at high altitude: A trend of elevated α-diversity has been reported across various species. This includes domesticated and wild animals such as Tibetan pigs and plateau crossbred pigs compared to lowland breeds (Luo et al., 2022; Zhao et al., 2023), rhesus monkeys at higher altitudes (Wu et al., 2020), plateau pikas (Li et al., 2019), high-altitude frogs (Niu et al., 2023, 2025), ungulates (Wang X. et al., 2022), and donkeys (Guo et al., 2022). A weak positive correlation with altitude was also observed in house mice (Suzuki et al., 2019).

Decreased or unchanged gut bacteriome diversity at high altitude: Contrasting results show decreased α-diversity in certain species, such as lizards (Zhang et al., 2018b), Tibetan chickens and other highland broilers compared to lowland poultry (Du et al., 2022; Bhagat et al., 2023). Other studies report no significant change, as seen in Sanhe heifers (Zhang et al., 2023b).

Complex or non-linear patterns: Some studies indicate that the relationship between altitude and diversity is not monotonic. For instance, a study on blind mole rats revealed that gut bacteriome α diversity peaked at mesoaltitudes (2000 m) (Solak et al., 2024).

Despite the variability in α-diversity outcomes, a highly consistent finding across nearly all studies is a significant shift in beta (β) diversity, which shows a positive correlation with elevation. This indicates that high-altitude exposure fundamentally restructures bacterial community composition.

Based on existing research, the seemingly contradictory findings regarding increased, decreased, or unchanged α-diversity in the gut bacteriome of high-altitude animals are actually attributable to multiple environmental factors such as hypoxia, low temperatures, intense ultraviolet radiation, and vegetation changes. These factors not only directly influence the host’s physiological and metabolic requirements but also indirectly alter its dietary composition. Therefore, we hypothesize that when high altitude leads to a more complex diet with higher fiber content (e.g., wild herbivores) and hosts are long-adapted native species, gut bacteriome α-diversity tends to increase to efficiently extract energy (e.g., Tibetan pigs, Tibetan donkeys, plateau pikas). While gut bacteriome diversity patterns vary across studies due to complex environmental interactions, host-specific ecology, and differing experimental designs, a consistent finding is the significant shift in beta diversity. This indicates that although the magnitude and direction of alpha diversity changes may fluctuate between cohorts, high-altitude exposure fundamentally reshapes bacterial composition and, consequently, the functional potential of the gut bacteriome. This reveals the most fundamental impact of the elevation effect: it does not simply increase or decrease bacteriome abundance, but fundamentally reshapes the species composition, ecological interactions, and functional potential of the gut bacteriome.

### 2.3 Effects of a low oxygen environment in highlands on the diversity of human gut bacteriome

Studies on humans also present a nuanced picture of how high-altitude hypoxia affects gut bacteriome diversity, influenced by factors such as duration of exposure, ancestry, and diet.

Increased diversity associated with adaptation: A higher α-diversity has been observed in the indigenous, long-term adapted Tibetan population compared to lowland Han Chinese (Lv et al., 2023). Furthermore, individuals developing Acute Mountain Sickness (AMS) upon ascending to high altitude showed a significant increase in α-diversity compared to their sea-level baseline (Wells et al., 2020; Ma et al., 2025).

Decreased or unchanged diversity: Conversely, short-term high-altitude exposure in Han Chinese migrants has been linked to a significant decrease in α-diversity (Jia et al., 2020). Other studies found no significant difference in diversity between Tibetan and Han populations on the plateau, or between these groups and lowland Han Chinese (Li and Zhao, 2015). Simulated normobaric hypoxia equivalent to 4,000 m in the PlanHab study also did not alter gut bacteriome diversity or composition (Šket et al., 2017, 2018).

The differences observed between high-altitude Tibetan populations and lowland Han Chinese may be more attributable to genetic factors and dietary variations. Long-term adaptation to the plateau has enabled Tibetan populations to develop unique hypoxia-related genes. These genes synergize with their traditional diet, centered on high-fiber barley and fermented dairy products, to shape and maintain a more stable, functionally adapted, and highly diverse gut bacteriome. Conversely, newly migrated Han populations from lowland areas experience short-term gut bacteriome changes primarily driven by acute hypoxic stress, often manifesting as reduced diversity or disruption. However, when modern lifestyles lead to highly homogenized diets across both groups, the convergent effects of this powerful recent dietary factor may mask deeper differences rooted in genetics and long-term adaptation. This could explain why some studies report no significant differences.

## 3 Altered composition of gut bacteriome in the low oxygen environment of plateau

Although microbial diversity under plateau hypoxia remains controversial, bacterial composition in hypoxic plateau environments appears to exhibit certain similarities. Studies indicate that hypoxic conditions may lead to overgrowth of anaerobic bacteria within the host gut (Moreno-Indias et al., 2015), decrease in the number of aerobic bacteria and increase in the number of specialized anaerobic bacteria in the gut bacteriome of rats under acute hypoxia (Maity et al., 2013). Soldiers stationed in highland areas found a 50-fold decrease in the number of aerobic bacteria and a 115-fold increase in the number of anaerobic bacteria in their intestines 15 days after reaching 3,505 m above sea level (Adak et al., 2013). Also in the investigation of house mice it was shown that strictly anaerobic bacteria were positively correlated with altitude, and facultative anaerobes, microaerophiles, and aerotolerant bacteria were all negatively correlated with altitude (Suzuki et al., 2019). The research by Bai et al. (2022) yielded similar results: at elevations of 4,000–5,000 m, aerobic bacteria decreased while anaerobic bacteria increased (Zhang et al., 2018a; Lv et al., 2023). Based on consistent evidence from multiple cross-species studies, high-altitude hypoxic environments induce alterations in gut bacteriome composition, characterized by a significant increase in anaerobic and facultative anaerobic bacteria abundance and a substantial decrease in aerobic bacteria. At high altitude, the microbial community becomes dominated by obligate anaerobes capable of efficient fermentation, which helps provide energy and maintain intestinal barrier integrity. Our analysis of the current literature reveals that findings regarding gut bacteriome alterations vary considerably across studies. These discrepancies are likely attributable to differences in study subjects, exposure protocols, dietary habits, and environmental conditions.

We have summarized and analyzed the current literature and found that the variation in bacterial bacteriome varies considerably from one literature to another, which may be due to different experimental subjects, treatment conditions, dietary habits, etc., However, differences in *Prevotella*, *Akkermansia*, and *Alipstipes* appeared in most of these literatures. The enrichment of *Prevotella*, or the ratio of *Bacteroidetes* to *Prevotella*, has been proposed as a potential biomarker for the metabolic profiling of intestinal microbes (Costea et al., 2018; Precup and Vodnar, 2019). The ratio of *Mycobacterium* to *Prevotella* as a possible marker for identifying individuals who may be more susceptible to the effects of low oxygen at altitude (Pan et al., 2022). However, it is interesting to note that the abundance of *Prevotella* under the plateau has shown very different results in different studies.

### 3.1 Prevotella

Short-term/acute exposure studies: Firstly, under acute plateau hypoxia, it has been shown that the abundance of *Prevotella* is reduced in the hypoxic group of rats and the *Bacteroides* to *Prevotella* ratio is increased (Pan et al., 2022; Wang et al., 2023a). However, in another study that looked at the dynamics of gut bacteriome over a 5 weeks period of exposure to low oxygen at altitude, it was found that *Prevotella* was rapidly enriched in low oxygen, while *Bacteroides* fluctuated dynamically, and it was suggested that there was a dynamic competition between *Bacteroides* and *Prevotella* (Han et al., 2022). Similarly, in a 30-day study on the dynamics of gut microbes in plateau hypoxia, it was found that the abundance of *Prevotella* was found to increase in the high altitude group at all times except day 7, and that the most significant and long-lasting changes in the gut bacteriome of rats in the two different altitude hypoxia groups were found on day 7 (Bai et al., 2022). Higher *Prevotella* abundance was also found to be associated with more severe AMS symptoms in an acute plateau hypoxia model in mice (Pan et al., 2022).

Wildlife/chronic exposure studies: In a study examining the gut bacteriome of pikas at different elevations, *Prevotella* showed a more pronounced enrichment in the gut as elevation increased (Li et al., 2016). However, another study on high-altitude blind mole rats did not detect characteristic *Prevotella* species, inconsistent with findings from other research. This discrepancy may stem from population variations caused by differences in host species (Solak et al., 2024).

Human population studies: A study examining gut bacteriome in residents across different altitudes on the Qinghai-Tibet Plateau found that *Prevotella* constitutes the core microbial community in the Tibetan population (Lan et al., 2017). Another study comparing different high-altitude resident groups found that at the same elevation (3,600 m), the Tibetan indigenous population exhibited higher relative abundance of *Prevotella* compared to Han Chinese immigrants (Li et al., 2016). Li et al.’s (2016) study similarly demonstrated this discrepancy, hypothesizing that differing dietary habits between Tibetan and Han populations may account for the disparity. *Prevotella* exhibited a negative correlation with energy intake, with Tibetans consuming more energy than Han individuals (Li and Zhao, 2015).

However, its health effects are still highly controversial and *Prevotella* is receiving increasing attention. Studies have shown that *Prevotella spp.* can improve glucose metabolism stimulated by prebiotic intake and are beneficial microorganisms (Kovatcheva-Datchary et al., 2015). These could explain the gastrointestinal stress response in the gut after hypoxic exposure at altitude. Also the abundance of *Prevotella* is not only associated with symptoms of rheumatoid arthritis, but also with people at high risk of developing the disease (Alpizar-Rodriguez et al., 2019; Wells et al., 2020). Also *P. copri*-specific antibodies can mediate pro-inflammatory TH17 and TH 1 cellular immune responses in patients with rheumatoid arthritis. Moreover, Th17 cells were stable in the gut but not in the gut of germ-free mice, suggesting that this subpopulation is produced in response to gut bacteriome and that colonization by *Prevotella* can induce a high production of Th17 cells in the mouse colon (Maeda et al., 2016). At the same time, *P. copri complex* was able to break down plant polysaccharides and host-derived mucins, but not dietary-derived animal polysaccharides, which may lead to increased intestinal permeability (Wright et al., 2000; Fehlner-Peach et al., 2019).

It was shown that *Prevotella spp* was able to utilize arabinoxylan and oligofructose to produce short-chain fatty acid propionates *in vitro* (Chen et al., 2017). Another study indicates that *Prevotella* colonization in the gut triggers metabolic alterations in the bacteriome, leading to reduced production of short-chain fatty acids (SCFAs, particularly acetate) and IL-18. This, in turn, exacerbates intestinal inflammation and may induce systemic autoimmune responses (Iljazovic et al., 2021). The discrepancy between these two findings may stem from differing intestinal microenvironments. In healthy intestines maintained by high-fiber diets, *Prevotella* effectively breaks down complex polysaccharides, primarily generating acetate and succinate to directly increase SCFAs (Jiang et al., 2022). This process positively impacts host metabolism and immune regulation. However, under pathological conditions, abnormal proliferation of *Prevotella* disrupts microbial balance, leading to adverse effects: on one hand, it may competitively inhibit the growth of bacteria producing other essential SCFAs; on the other hand, accumulation of its metabolic byproduct succinate may not be effectively metabolized, thereby exacerbating intestinal inflammation (Jiang et al., 2022). SCFAs as primary metabolites produced by gut bacteriome fermentation of dietary fiber, play a crucial physiological regulatory role in high-altitude adaptation. Their mechanisms of action can be summarized across three interconnected dimensions: At the metabolic level, SCFAs provide essential energy substrates for host tissues, particularly colonic epithelial cells, partially compensating for reduced mitochondrial oxidative phosphorylation efficiency caused by hypoxia and helping maintain basal energy homeostasis (Chen S. et al., 2025). At the barrier function level, SCFAs (particularly butyrate) significantly enhance intestinal epithelial barrier integrity by upregulating tight junction proteins (e.g., occludin and ZO-1) and promoting mucin secretion. This effectively reduces the risk of increased intestinal permeability and subsequent endotoxin translocation potentially induced by high-altitude hypoxia and low pressure (Zhou et al., 2017; Xiao J. et al., 2024). At the immunoregulatory level, SCFAs promote the differentiation and function of regulatory T cells through epigenetic mechanisms such as histone deacetylase inhibition, while simultaneously suppressing the excessive activation of pro-inflammatory signaling pathways like NF-κB. This helps buffer against excessive immune responses triggered by high-altitude environmental stress (Yang et al., 2020). Thus, SCFAs exert beneficial effects on host physiological adaptation to high-altitude environments. Conversely, reduced SCFAs production due to dysbiosis may correlate with impaired energy metabolism, barrier dysfunction, and elevated systemic inflammation, collectively exacerbating the pathophysiological processes underlying poor high-altitude adaptation.

### 3.2 Akkermansia

Currently there are only three species in the genus *Akkermansia: A. muciniphila*, *A. glycaniphila*, and *A. biwaensis*. *A. muciniphila* is the first species of the warty bacteriome.

Short-term/acute exposure studies: A study on C57BL/6J mice revealed a significant increase in *Akkermansia* abundance following 24 h of acute exposure to high altitude (5,500 m) (Wang F. et al., 2022). SD rats exposed to 5,500 m for 3 days also exhibited the same results (Wang et al., 2023a).

Wildlife/chronic exposure studies: A study on gut bacteriome in plateau frogs reveals a significant enrichment of *Akkermansia*. However, another study on blind mole rats found that *Akkermansia* was present only in low-altitude populations and was not detected in high-altitude animals (Solak et al., 2024). Research by Liu et al. (2020) indicates that *Akkermansia* abundance in Tibetan wild asses is significantly higher than in domestic donkeys.

Human population studies: A survey study showed that the intestinal bacterial, bacteriome and virulence groups of 18 subjects living at high altitude (HA) and 30 subjects living at sea level (SL) were sequenced by deep whole-macro-genome sequencing. The findings revealed significant compositional and functional differences in the gut bacterial, fungal and viral communities between the two groups. The results of the study showed that *A. muciniphila* was significantly enriched in the high altitude population (Xiao Z. et al., 2024).

The seemingly contradictory findings regarding the abundance of *A. muciniphila* in high-altitude regions across two studies do not represent a genuine contradiction. Rather, they reflect the high dependence of bacteriome research on specific “host-environment-diet” ecological contexts. At the host level, different species—such as acutely exposed mice, chronically adapted blind mole rats, and humans residing on plateaus—exhibit markedly distinct gut physiology and immune states. At the environmental level, short-term hypoxic stress may trigger temporary increases in protective bacteriome (e.g., *A. muciniphila*), while long-term adaptation may lead to structural reconfiguration of bacteriome communities. Regarding dietary composition, high-fiber traditional diets (like the barley and fermented dairy products of Qinghai-Tibet Plateau residents) may provide abundant substrates, while specialized feeding habits (such as the mole rat’s reliance on plant rhizomes) may alter its colonization environment. More importantly, during prolonged adaptation, hosts may leverage functional redundancy or niche replacement mechanisms to utilize other bacteriome groups for similar mucosal protection and metabolic regulation functions.

*A. muciniphila* is a gram-negative anaerobic bacterium belonging to *Verrucomicrobia*. *A. muciniphila* was isolated and enriched from human feces, where mucin serves as its primary carbon and energy source. *A. muciniphila* are mucin-degrading bacteria, but by promoting the differentiation of secretory IECs, mucus production is instead increased. Mucus covers the outer intestinal epithelial cell layer and acts as a physical protector. *A. muciniphila* are negatively associated with a variety of diseases (Hasani et al., 2021; Rodrigues et al., 2022; Xie J. et al., 2023; Xie S. et al., 2023), *A. muciniphila* is ubiquitous in the human gastrointestinal tract and is abundant at all stages of life. Not only is it present with in the feces and mucous membranes, it has been shown to be present in the oral cavity and small intestine. *Amuc_1100* is an outer membrane heat stabilizing protein secreted by *A. muciniphila*, which may mediate the probiotic effects of *A. muciniphila* (Anhê and Marette, 2017), *A. muciniphila* may function through this protein (Chen et al., 2022). *A. muciniphila* protects the integrity of the intestinal mucosal barrier, which is strongly correlated with intestinal acylglycerol levels, and Everard et al. (2013) demonstrated that *A. muciniphila* treatment increased levels of 2-palmitoylglycerol (2-PG), 2-arachidonic acid ester acylglycerol (2-AG), and 2-oleoylglycerol (2-OG), which protects the intestinal mucosa. The study showed that feeding live *A. muciniphila* counteracted the reduction of the mucus layer caused by a high-fat diet (Scherer and Buettner, 2009; Muccioli, 2010; Alhouayek et al., 2011; Everard et al., 2013; Kim et al., 2021). It has been reported that *A. muciniphila* is reduced by approximately 17% in SD rats exposed to hypoxia and that treatment of animals with synbiotics (a mixture of prebiotics and probiotics) improves mucosal barrier integrity and reduces inflammation (Mishra and Bakshi, 2023).

### 3.3 Alistipes

**FIGURE 2:**
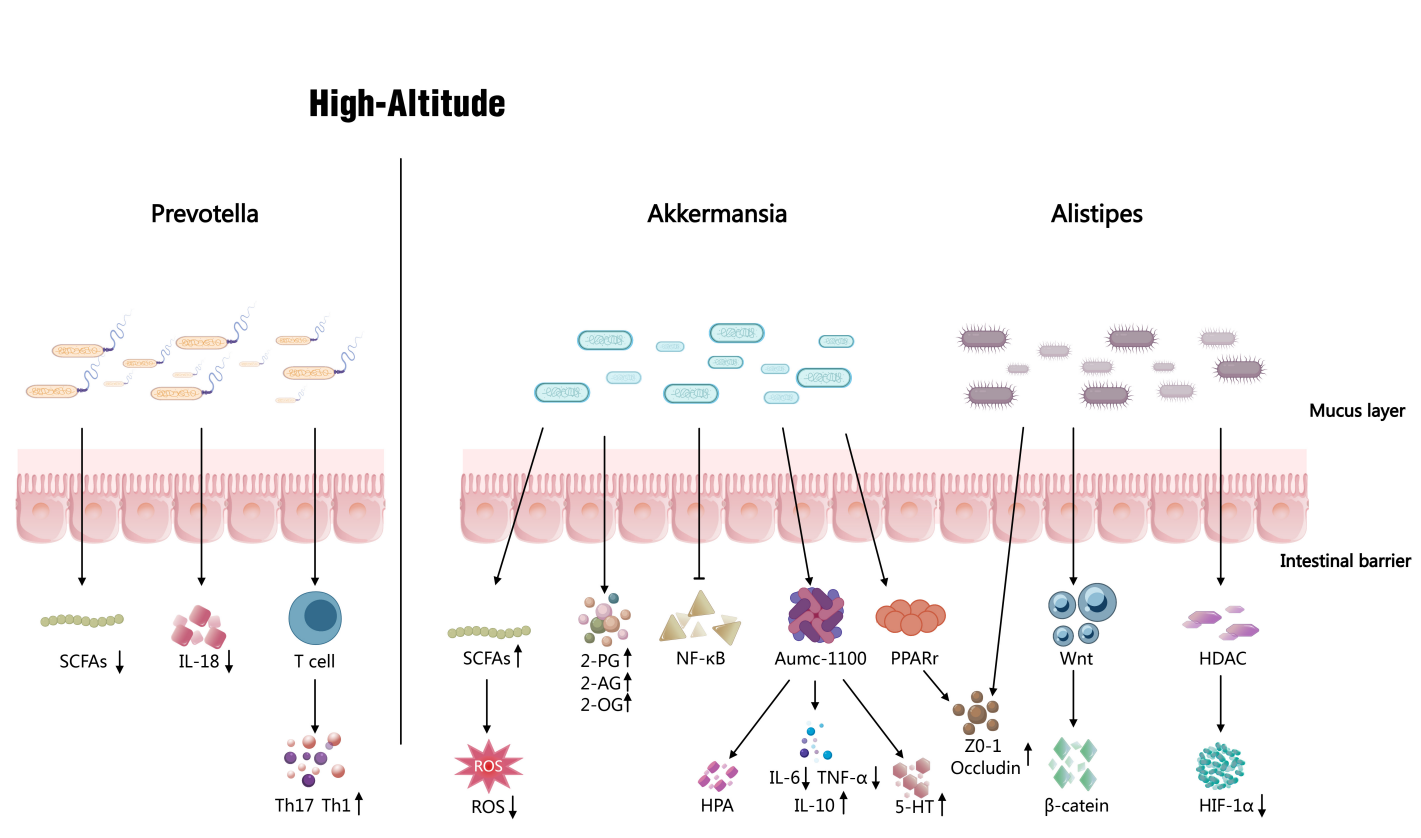
Expression of *Prevotella*, *Akkermansia*, and *Alistipes* in hypoxic exposure and mechanisms of gastrointestinal mucosal regulation.

*Alistipes* is a relatively new subclade of the phylum Mycobacterium and is a Gram-negative bacterium. *Alistipes* are anaerobic bacteria that are found primarily in the gut bacteriome of the healthy human gastrointestinal tract (Shkoporov et al., 2015). The species currently found in the genus are *Alistipes finegoldii, Alistipes putredinis, Alistipes onderdonkii, Alistipes shahii, Alistipes indistinctus, Alistipes Alistipes onderdonkii, Alistipes shahii, Alistipes indistinctus, Alistipes senegalensis, Alistipes timonensis, Alistipes obesi, Alistipes ihumii, Alistipes inops, Alistipes megaguti, Alistipes provencensis*, and *Alistipes massiliensis*. *Alistipes* are significantly enriched under low-pressure hypoxia, with marked alterations in response to changes in oxygen concentration, possibly showing an opposite trend to *Prevotella*, and their interaction contributes to the dynamics of the intestinal bacteriome (Han et al., 2022). *Alistipes* are also thought to be associated with intestinal inflammation, and one study showed that when mice with DSS-induced colitis were tube-fed with *A. finegoldii*, it was able to reduce the severity of colitis (Dziarski et al., 2016). Although *Alistipes* play an active role in colitis, in contrast, *Alistipes* have been shown to be pathogenic in disorders such as anxiety, myalgic encephalomyelitis/chronic fatigue syndrome, depression, Pervasive Developmental Disorder Not Otherwise Specified (PDD-NOS) and Colorectal cancer (CRC) (Bangsgaard Bendtsen et al., 2012; De Angelis et al., 2013; Frémont et al., 2013; Jiang et al., 2015; Polansky et al., 2015; Strati et al., 2017). And is elevated in chronic fatigue syndrome, irritable bowel syndrome and depression (Naseribafrouei et al., 2014). It has been suggested that these high levels of *Alistipes* may be a potential cause of hypoxia-induced intestinal disease (Frémont et al., 2013; Figure 2).

Short-term/acute exposure studies: Zhang et al. (2018a) demonstrated that after 30 days of exposure to a hypoxic environment at high altitude, the abundance of *Alistipes* bacteria in the gut of mice increased, consistent with the findings Pan et al. (2022).

Human population studies: An analysis of gut bacterial communities in indigenous Tibetans and Han Chinese immigrants revealed significantly higher levels of *Alistipes* in Tibetans compared to Han Chinese (Ma et al., 2021).

For these findings, we attribute the results to different altitudes and different times of hypoxic treatment, and that all of these acute treatments lasted for a maximum of 5 weeks, whereas in an Fecal microbiota transplantation (FMT) report on plateau hypoxic mice, it was shown that fecal transplants from mice exposed to plateau hypoxia for 4 weeks to antibiotic cocktail-treated mice kept under normoxic conditions, similarly caused intestinal damage in mice, suggesting that 4 weeks of hypoxic exposure did not result in complete plateau-adapted changes in the gut bacteriome of mice (Wang F. et al., 2022). Therefore, we believe that the differences in these results may be due to the fact that it takes a longer period of time for the gut microbiological changes in mice to adapt to the plateau environment, and that the microbial dynamics during this period of time led to the differences in the results of the previous studies. Therefore, in order to better elucidate and understand the changes in gut microbes at plateau, it may be necessary to analyze the dynamics of gut microbes in experimental animals over a longer period of time, and to further analyze the effects of different altitudes and study subjects on the changes in gut microbes.

## 4 Other factors contributing to changes in gut bacteriome

The high-altitude environment exerts a certain influence on dietary patterns, which may directly shape unique gut bacteriome profiles. The differences in the gut bacteriome of long-term highland-dwelling animals may be related to diet (Ley et al., 2006; Duncan et al., 2007; Muegge et al., 2011; Walker et al., 2011; Wu et al., 2011). Research has shown that the gut bacteriome can respond rapidly to a changing diet. Food composition has a key impact on the gut bacteriome, influencing its compositional richness and diversity. On the one hand, high intake of animal proteins, saturated fats, sugar and salt stimulates the growth of pathogenic bacteria, which can impair beneficial bacteria and damage the gut barrier. In addition, complex polysaccharides and plant proteins may be associated with an increase in the number of beneficial bacteria, thus stimulating the production of SCFAs. In addition, omega-3s, polyphenols and micronutrients appear to have the potential to confer health benefits by modulating the gut bacteriome (Rinninella et al., 2019). Studies have shown that short-term consumption of diets consisting exclusively of animal or plant products alters microbial community structure and alters inter-individual differential microbial gene expression. Animal-based diets increased levels of bile-resistant microorganisms (*Alistipes*, *Bilophila*, and *Bacteroides*) and decreased levels of the thick-walled phylum Bacteroides. Microbial activity reflects differences between herbivory and carnivory Mammals (Muegge et al., 2011). Results show that the gut bacteriome can respond quickly to altered diets (David et al., 2014). Analysis of the gut bacteriome of Tibetans residing long-term in Lhasa (3,600 m) and Han Chinese from lowland regions revealed that most Han individuals exhibit a Bacteroides-dominant gut type, while most Tibetans display a Prevotella-dominant gut type (Li et al., 2016). This difference may stem from distinct dietary patterns: Han diets are characterized by low fiber, high animal protein, and high fat intake, whereas Tibetan diets in high-altitude regions are more associated with high fiber and high carbohydrate consumption.

**FIGURE 3:**
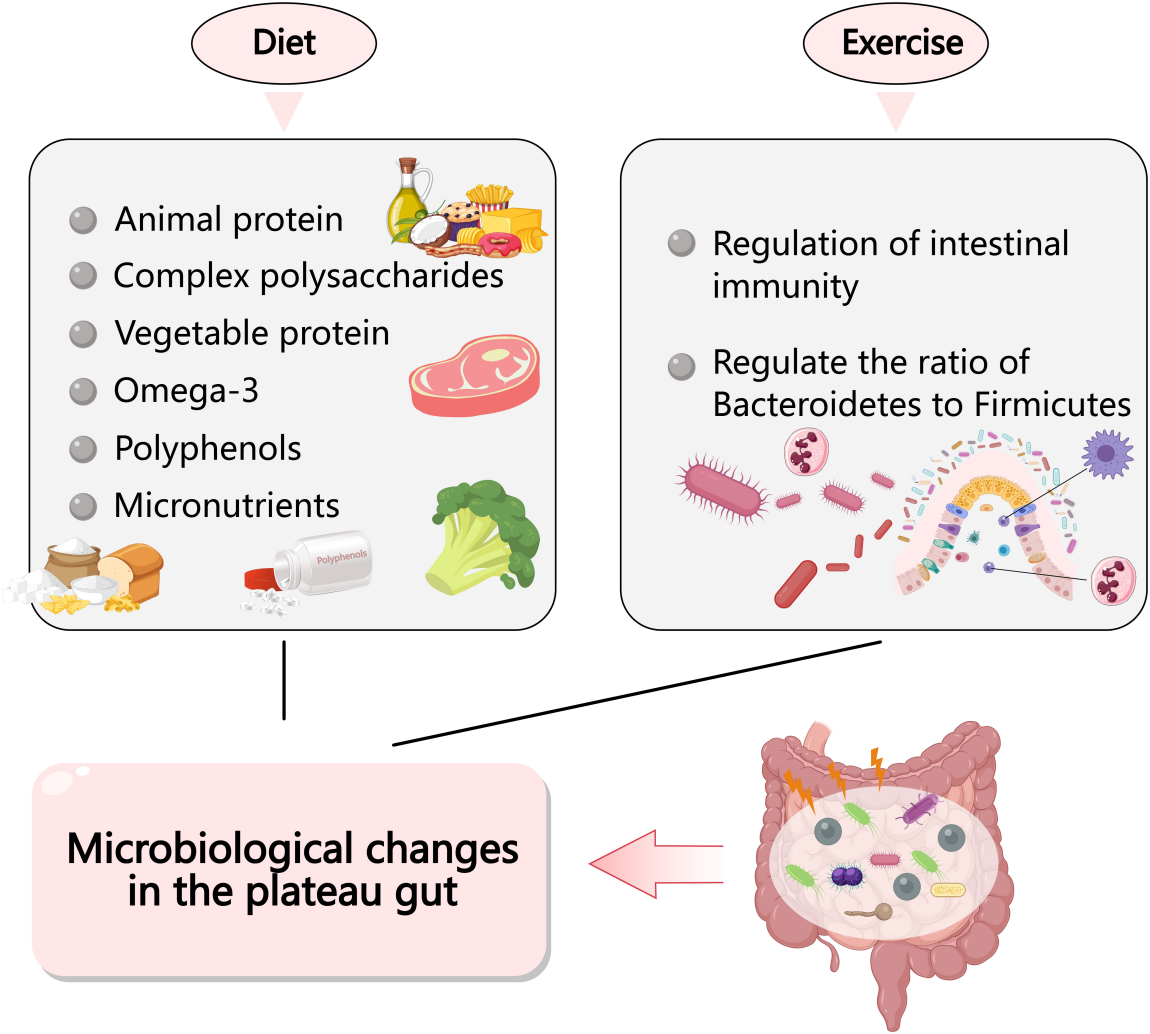
Other influences on gastrointestinal microecology in the highlands.

The hypoxic environment of high altitudes profoundly alters the physiological load and metabolic consequences of physical activity, thereby indirectly regulating the gut bacteriome. Under hypoxic conditions, physical activity of the same intensity results in greater physiological stress and energy expenditure. Several studies have shown that physical activities such as exercise and sport can alter gut bacteriome composition (Monda et al., 2017; Mailing et al., 2019). Exercise can prevent weight gain and obesity and alter the ratio of *Bacteroidetes* and *Firmicutes* in mouse models (Evans et al., 2014; Campbell et al., 2016). A study by Allen et al. (2018) reported different effects of spontaneous and mandatory training on changes in the gut bacteriome of mice with inflammatory injuries such as ulcerative colitis. These changes may be related to intestinal immune function and lead to colitis. Exercise can lead to an increase in certain types of bacteria, such as lactic acid-producing bacteria (for example, *Bifidobacterium* and *Lactobacillus*), They can regulate mucosal immunity and prevent pathogen invasion, or Blautia coccoides and Eubacterium rectale convert lactic acid into butyrate (Queipo-Ortuño et al., 2013; Forsythe et al., 2014). In addition, exercise can determine qualitative and quantitative changes in the composition of the human microbial bacteriome, and athletes have greater bacteriome biodiversity. Exercise may alter the abundance of several taxa and species according to body mass index status: in lean subjects, exercise increased fecal bacillus species and decreased bacillus-like species, whereas the opposite was true in obese subjects; butyric acid-producing taxa also increased in lean subjects, but these effects were transient and disappeared during the subsequent sedentary washout period (Allen et al., 2018; Cronin et al., 2018; Figure 3). However, under the hypoxic conditions of high altitudes, these exercise-induced changes may be amplified or distorted (Bigard et al., 1991). On one hand, moderate, regular activity reinforces the beneficial microbial effects by enhancing intestinal blood flow and reducing hypoxia-induced damage to the intestinal barrier, thereby aiding the host’s adaptation to the environment. On the other hand, acute intense exercise combined with hypoxic stress may trigger a strong systemic inflammatory response and increase the risk of intestinal ischemia. This could temporarily suppress butyrate-producing bacteria and disrupt bacteriome balance, potentially contributing to the onset of acute mountain sickness (Álvarez-Herms et al., 2025). These hypotheses require further research for definitive confirmation.

## 5 Conclusion

**FIGURE 4:**
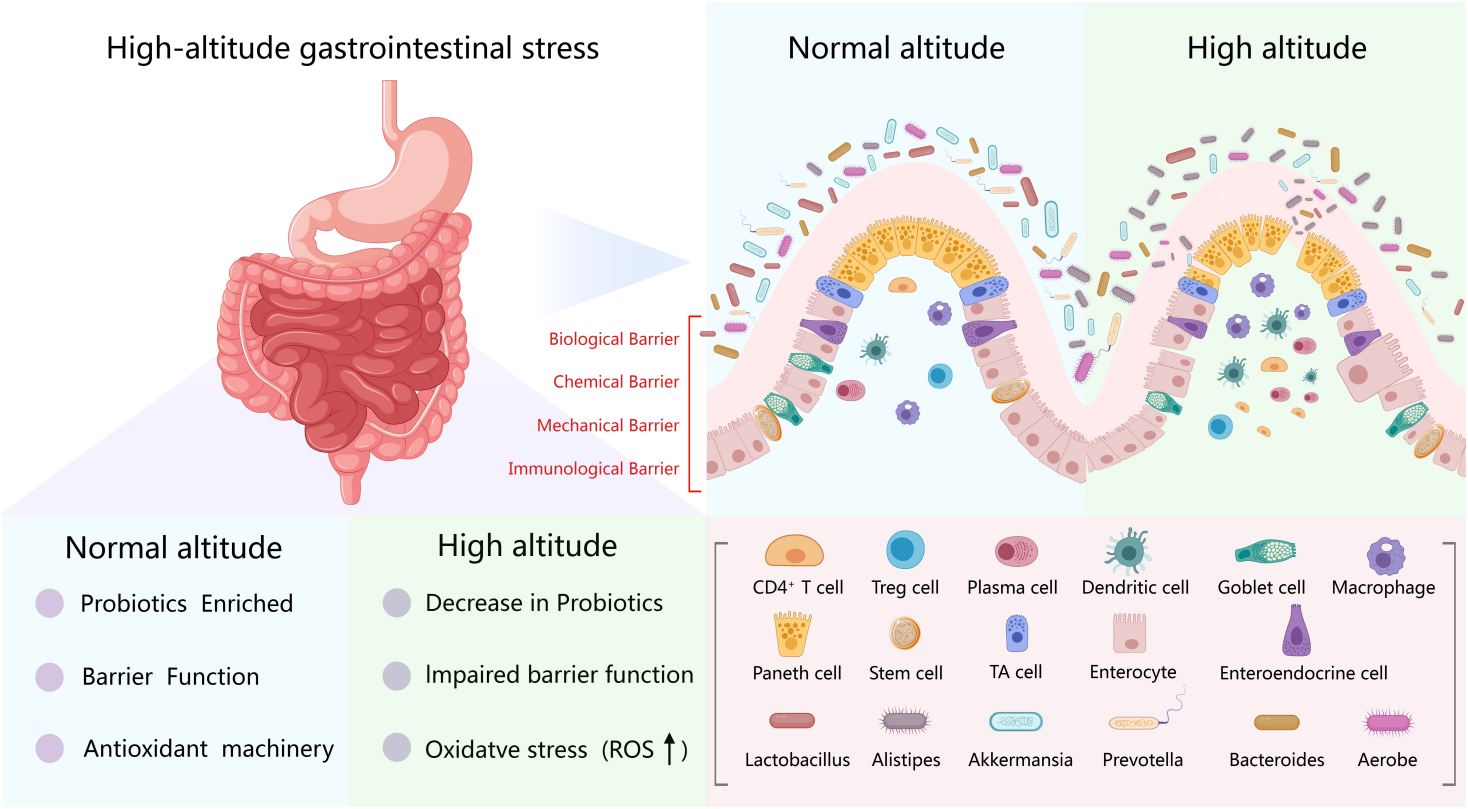
Schematic diagram of gut bacteriome changes in the plateau environment.

In summary, this review synthesizes compelling evidence demonstrating that prolonged exposure to high-altitude hypoxia fundamentally reshapes the composition, diversity, and functional potential of the gut bacteriome in both animals and humans. A key established consensus is the consistent and significant shift in beta diversity across nearly all studies, indicating a profound reorganization of bacteriome community structure in response to elevation. While trends in alpha diversity are more variable that showing increases, decreases, or non-linear patterns depending on the host species, diet, and study design, this variability itself underscores the complexity of host-bacteriome-environment interactions at altitude (Table 1, Figure 4).

**TABLE 1:** Changes in gut bacteriome abundance in different plateau environments.

Animal models	Study design	Microbial diversity
C57BL/6 mice	24 h at 5,500 m	α+* β w/
BALB/c mice	7 days at 6,000 m	α + * β w/
Healthy men	21 days at 4,000 m	α 0 β w/o
Male Wistar rats	28 days at 5,000 m	α 0 β w/
SD rats	28 days at 5,000 m	α+* β w/
C57BL/6 mice	4 weeks at 4,000 m	α+* β w/
Short-term plateau living Han Chinese population	4 days at 4,000 m	α−* β w/
BALB/c mice	30 days at 5,000 m	α 0 β w/
Tibetan population	>3,000 m	α+* β w/
Tibetans	2,800∼4,500 m altitude	α+* β w/
Tibetan pigs	3,500–3,700 m	α+* β w/
Plateau ternary crossbred commercial pigs	3,500–3,700 m	α+* β w/
Altitude rhesus	>3,000 m	α+* β w/
Tibetan chickens	3,572 m	α−* β w/
Lizards	2,900–4,250 m	α−* β w/
Blind mole rats	1,000–3,000 m	α+* β w/
Plateau pikas	3,100–4,300 m	α+* β w/
Frogs	3,730 m	α+* β w/
Ungulates	2,700 m	α+* β w/
Donkey	>3,000 m	α−* β w/
Sanhe heifers	3,650 m	α 0 β w/

*, Significant 0, Not significant +, Increased diversity -, Decreased diversity w/, Diversity change observed w/o, No diversity change observed.

Critical gaps remain in the current literature. First, most human studies are cross-sectional, conflating the effects of hypoxia with dietary and genetic differences; controlled, longitudinal studies are needed to disentangle these factors. Second, the precise mechanistic pathways linking hypoxia to bacteriome ecology are still being elucidated, particularly the roles of host-derived metabolites (e.g., SCFAs, bile acids) and immune modulation. Third, there is a paucity of research integrating multi-omics data (metagenomics, metabolomics, host transcriptomics) to move beyond correlation and establish causative links between bacteriome shifts and host physiological outcomes.

The physiological and health implications of these bacteriome shifts are significant. The adapted gut bacteriome in high-altitude natives appears to be a co-evolved trait that enhances energy harvest from scarce resources, modulates systemic inflammation. Conversely, the dysbiosis observed in non-adapted individuals during acute exposure may contribute to the pathophysiology of altitude-related illnesses, such as acute mountain sickness. This positions the gut bacteriome not merely as a passive responder, but as an active mediator of hypoxic adaptation and maladaptation.

Future research should delve deeper into the mechanisms underlying gut bacteriomel changes in high-altitude environments. Beyond conducting longitudinal human intervention studies to track the dynamic adaptation of bacteriome, future investigations should integrate multi-omics approaches in rigorously controlled animal models to elucidate action mechanisms. Furthermore, expanding research scales to include non-mammalian models and high-altitude populations is essential for comprehensively understanding bacteriome adaptation to environmental conditions. Research in these areas will not only elucidate the role of gut bacteria in high-altitude biology but also provide new insights into the evolution of host-bacteriome symbiosis under environmental stress.
